# Meta-analytic approach to the accurate prediction of secreted virulence effectors in gram-negative bacteria

**DOI:** 10.1186/1471-2105-12-442

**Published:** 2011-11-14

**Authors:** Yoshiharu Sato, Akiko Takaya, Tomoko Yamamoto

**Affiliations:** 1 Graduate School of Pharmaceutical Sciences, Chiba University, Chiba 260-8675, Japan

## Abstract

**Background:**

Many pathogens use a type III secretion system to translocate virulence proteins (called effectors) in order to adapt to the host environment. To date, many prediction tools for effector identification have been developed. However, these tools are insufficiently accurate for producing a list of putative effectors that can be applied directly for labor-intensive experimental verification. This also suggests that important features of effectors have yet to be fully characterized.

**Results:**

In this study, we have constructed an accurate approach to predicting secreted virulence effectors from Gram-negative bacteria. This consists of a support vector machine-based discriminant analysis followed by a simple criteria-based filtering. The accuracy was assessed by estimating the average number of true positives in the top-20 ranking in the genome-wide screening. In the validation, 10 sets of 20 training and 20 testing examples were randomly selected from 40 known effectors of *Salmonella enterica *serovar Typhimurium LT2. On average, the SVM portion of our system predicted 9.7 true positives from 20 testing examples in the top-20 of the prediction. Removal of the N-terminal instability, codon adaptation index and ProtParam indices decreased the score to 7.6, 8.9 and 7.9, respectively. These discrimination features suggested that the following characteristics of effectors had been uncovered: unstable N-terminus, non-optimal codon usage, hydrophilic, and less aliphathic. The secondary filtering process represented by coexpression analysis and domain distribution analysis further refined the average true positive counts to 12.3. We further confirmed that our system can correctly predict known effectors of *P. syringae *DC3000, strongly indicating its feasibility.

**Conclusions:**

We have successfully developed an accurate prediction system for screening effectors on a genome-wide scale. We confirmed the accuracy of our system by external validation using known effectors of *Salmonella *and obtained the accurate list of putative effectors of the organism. The level of accuracy was sufficient to yield candidates for gene-directed experimental verification. Furthermore, new features of effectors were revealed: non-optimal codon usage and instability of the N-terminal region. From these findings, a new working hypothesis is proposed regarding mechanisms controlling the translocation of virulence effectors and determining the substrate specificity encoded in the secretion system.

## Background

Protein secretion and translocation into eukaryotic host cells are key processes in the virulence of pathogenic bacteria [1]. So far, six different secretion systems have been described for Gram-negative bacteria [2,3]. Among these, the type III secretion system (TTSS) is a representative apparatus that secretes and translocates virulence proteins out of bacterial cells. Representative models of pathogens using TTSS as the main secretion system are the animal pathogens *Salmonella, Yersinia*, and *Shigella *and the plant pathogens *Pseudomonas *and *Xanthomonas*. Since effector secretion is an important strategy for the virulence of these bacteria, many research groups in the bacterial infection field have made great efforts to identify secretion substrates [4-8]. In these studies, elaborate proteomic and genetic screening methods have been established and many effectors have been identified by genome-wide high-throughput screens such as the translocation assay of CyaA-fused proteins from libraries of transposon-mediated random insertions in the genome [9]. However, the effector repertories, even for deeply investigated pathogens such as *Salmonella*, have had to be revised continuously [10,11]. Moreover, considering the complexity and elaborated infectious strategy of *Salmonella*, there may be more effectors yet to be detected. This situation indicates that the utility of the established genome-wide experimental screenings is limited and that new approaches will be necessary to develop a complete catalogue of effectors. Bioinformatics-assisted effector identification is a promising alternative approach. Previous studies have successfully identified novel effectors by using homology-search-based screening [7] or feature-extraction-based approaches such as promoter motif search and analysis of N-terminal amino acid composition bias [12]. Furthermore, recent progress in sequencing technology has enabled whole genomes to be sequenced quickly, at reasonable cost [13]. In fact, the genomes of many pathogenic bacteria have been sequenced and continue to be sequenced at a growing speed, enabling bioinformatics-based identification of virulence effectors for an expanding number of such bacteria. This supports the development of various prediction tools. However, accurate prediction of TTSS substrates is a very challenging problem because no clear consensus motif has been defined for these substrates. In addition, the secretion mechanism is still largely uncharacterized at the molecular level, as exemplified by the absence of co-purified crystal structures of the effector and its translocator. Homology searching is a straightforward method of sequence-based screening [7]. However, effector genes generally evolve rapidly to adapt to different hosts [14] or to escape from a severe immune response by the host, which makes homology-based approaches difficult. Moreover, the homology search approach alone cannot identify novel effectors. As another bioinformatics approach, machine-learning-based methods have been recently developed. Most of these approaches implement the position-specific amino acid composition profiling [15] or naïve Bayes approach [16] to capture a weak signal and composition bias in the N-termini of effectors. Enrichment of Thr/Ser and depletion of Glu/Asp residues in the N-terminal region is a feature of TTSS substrates commonly observed for a wide range of organisms that utilize TTSS [16]. Other machine-learning techniques, using support vector machine (SVM) [17,18] or artificial neural network [19] approaches, have also been developed. In these approaches, many more feature parameters are included in addition to sequence motif and composition bias, *e.g.*, GC content, secondary structure prediction, and phylogenetic profiling. Using these tools, it has been reported that low GC content, atypical phylogenetic relationships showing characteristics of horizontal gene transfer, and enrichment of coiled regions with high solvent accessibility are useful for discriminating effector genes from non-effectors, although findings regarding the N-terminal flexibility of the secretion substrate are controversial [15,16,19]. These prediction tools have achieved a certain degree of accuracy and, combined with experimental proteomic analysis, have successfully identified novel effectors [10]. However, none of these tools has achieved sufficient accuracy for genome-scale identification as a sole screening device owing to the high rates of both false positives and false negatives. The ultimate goal of a prediction system is to produce an accurate effector candidate list that could help increase the efficacy of gene-directed experimental verification. To satisfy this demand, true positives must be enriched in the top-20 to -30 ranking of the whole genome prediction. However, as one example, if existing prediction tools such as SVM-based Identification and Evaluation of Virulence Effectors (SIEVE) [17], BPBAac [15] and Effective-T3 [16] were applied to all the genes of LT2, the list of the top 20 in the prediction ranking would include only two to five known effectors. Under this situation, experimentalists spend much labour to identify novel effectors by gene-targeted verification based on the candidate list predicted by the existing tools. This also suggests that some characteristics encoded in the TTSS substrates are still undiscovered. In this study, we propose a refined pipeline to predict secreted virulence proteins that is based on a combination of a machine learning approach that extracts discrimination features from amino acid sequences, nucleotide sequences and phylogenetic analyses, and data mining of gene expression databases. We confirmed that the optimized prediction system outperformed pre-existing prediction tools and that the prediction was accurate enough to conduct efficient gene-directed experimental verification. We also discuss previously unidentified or uncharacterized features of the virulence effectors, which were suggested through the refinement process of the prediction system.

## Results and discussion

### Dataset construction and prediction pipeline

In this analysis, we constructed a new approach for predicting effectors from discrimination features derived from the nucleotide and amino acid sequences and from DNA microarray experimental data. In our prediction system, a meta-analytic approach was adapted, beginning with a machine-learning-based discriminant analysis followed by coexpression analysis and other simple criteria-based filtering. To assess the accuracy of our system, a representative model organism was selected, *Salmonella enterica *serovar Typhimurium LT2 (hereafter denoted LT2). Another well-studied plant pathogen, *Pseudomonas syringae *DC3000 (hereafter DC3000), was also selected to test the wider feasibility of our system. As a gold-standard set of positive examples, *i.e.*, known effectors, 40 and 28 effectors from LT2 and DC3000, respectively, were collected from the literature and from our recent experimental results (See Additional file 1: Supp_Table_knownEffector.xls). All other non-effector genes were treated as negative examples and test samples for novel effector screening. We noticed that the translation initiation site of one known effector in LT2 was incorrectly annotated in the Kyoto Encyclopedia of Genes and Genomes (KEGG) database. Hence, we re-annotated the open reading frame (ORF) positions in the set of LT2 as described in the Methods section.

### Statistics regarding representative discrimination features (classifiers) in the support vector machine portion of the analysis

In the first part of our new effector screening approach, several new features were introduced into the SVM-based discriminant analysis. Statistical information regarding these features, together with "GC content" values for the discriminative features, is shown in Table 1. We confirmed that the average GC content is significantly lower in the known effector group than in the proteome in general. This may be due to the alien origin of effector genes (*i.e.*, genes acquired by horizontal transfer), as suggested by many previous studies [16,17,20,21]. In addition to GC content analysis, we also estimated the codon adaptation index (CAI) [22].

**Table 1:** Statistics for features used in the SVM part of discriminant analysis

	Known effectors (n = 40)		Proteome (n = 4510)	
	
	Average	Standard deviation	Average	Standard deviation
GC	**0.43**	± 0.05	0.52	± 0.05
CAI	**0.57**	± 0.04	0.68	± 0.06
N-terminal instability	**13.35**	± 6.67	3.97	± 5.52
Molecular Weight	40.79	± 19.49	34.53	± 24.50
Charge	**-8.83**	± 11.08	-2.62	± 12.66
pI	**6.08**	± 1.51	7.04	± 1.88
Instability index	**42.21**	± 8.57	37.77	± 10.83
Aliphatic index	**84.94**	± 10.53	95.32	± 17.25
GRAVY score	**-0.31**	± 0.25	-0.06	± 0.44
dN/dS	**0.42**	± 0.30	0.20	± 0.31

Parameters with statistically significant difference between known effectors and the proteome (Student's t-test, p-value < = 0.05) are shown in bold.

The CAI represents how codon usage of a given gene is optimized for effective translation, which was introduced by Sharp *et al. *[22]. It has been revealed that there is a selection pressure on the synonymous site, in which the nucleotide substitution does not cause the amino acid change. The selection pressure produces the codon usage bias in the synonymous site. In enteric bacteria, synonymous codon bias increases with gene expression levels [23]. This has been thought to be due to selection in favor of efficiently translated codons [24]. Each amino acid is encoded by one to six codons and each codon is associated with anti-codon tRNA. Since there is a variation for the copy numbers of tRNAs, the codon corresponding to the highly expressed tRNA is thought to have translational advantages in terms of rate and accuracy. Hence, the codon usage tends to be optimized in the highly expressed genes such as ribosomal proteins and chaperones. The CAI value tells us important biological implications related to translation. Recently, it has been suggested that the codon usage of the Sec dependent substrates tends to be non-optimal (*i.e.*, low CAI) [25,26]. In this study, we estimated the CAI values for known effectors and those for proteome of *Salmonella*.

The values were lower in the group of effectors, which may be because the codon usage of horizontally-acquired genes is generally not optimized at the time of transfer. As expected, the CAI values in the effector group were lower than those of the proteome in LT2. Although the difference is likely to stem from the same source as in low GC content, *i.e.*, the alien nature of the effectors, the degree of difference in CAI values (Student T-test p-value = 0) is greater than the difference in GC contents (Student T-test p-value = 6.66 × 10^-6^). Therefore, the use of CAI in the SVM analysis is expected to refine the overall accuracy of the discriminant analysis. As for the N-terminal instability index, many researchers have reported that the predicted secondary structure elements (coil, alpha helix, beta sheet) showed enriched coil regions in the N-termini of effectors [16-19]. In the present study, we estimated N-terminal instability through POODLE-S, a program that considers the context of a given region in calculating a score [27]. The index also showed a significant difference between members of the effector group and those of the proteome in general. Furthermore, physicochemical parameters estimated from the amino acid sequence by the ProtParam program also showed differences between the two groups. To summarize the ProtParam features, the effectors were likely to be unstable, less aliphatic and hydrophilic. These tendencies were also observed for known effectors of DC3000 (See Additional file 2 Supp_Table_StatDC3000.xls). As for the charge and pI parameters, the values showed opposite relationships between LT2 and DC3000 (Table 1 and Additional file 2 Supp_Table_StatDC3000.xls). The genes in the effector group have relatively negative and low pI values in LT2, whereas effectors in DC3000 have relatively positive and high pI values, compared with those of the proteome in general. This may reflect differences in environmental conditions in which the effectors function. The rate of effector evolution was estimated to be faster than that of housekeeping genes, as reported in previous studies [28,29].

### Predictive power of the SVM-based discriminant analysis

We assessed the results from the SVM analysis by area under the curve (AUC) and average rank of known effectors (RANKavg) in the testing set (Table 2), as described in the Methods section. The values were averaged over 10 randomly selected validation sets (Set1~Set10) to eliminate the effect of selection bias for positive and negative examples in the training set. The AUC for the SVM using all parameters (ALL in Table 2) was estimated to be 0.993, which was greater than that of published tools, *e.g.*, 0.97 for SIEVE and 0.89 for Effective-T3. Due to differences in validation criteria, the assessment of performance by simply comparing AUC values is problematic; however, a clear enrichment of known effectors in the top ranks was observed. Since ten to twenty candidates were chosen for laboratory-based experimental verification, the average number of true positives in the top 20 was estimated to assess the prediction accuracy. In our all-parameter model, 9.7 true positives out of 20 known effectors tested were successfully ranked for the top 20. This can be compared with 5.0 true positives on average by BPBAac prediction, which showed the best performance among the BPBAac, SIEVE, and Effective-T3 tools, in our validation. It is noteworthy that the BPBAac requires no information about known effectors, so it can be applied to any genome with no known effectors. We also examined the pan-genomic feasibility of our system through cross-species prediction, *i.e*. training using information on known effectors from distantly related bacteria. As a result, the AUCs for cross-species prediction were 0.989 and 0.981 for the LT2-to-DC3000 model and the DC3000-to-LT2 model, respectively (See Additional file 3 Supp_Table_CrossPred.xls). In the DC3000-to-LT2 prediction, the average number of true positives for 20 known effectors tested was estimated to be 4.4. This value is slightly lower than the BPBAac value of 5.0, which showed the best score among the existing tools. However, the training set for BPBAac includes almost all of the known *Salmonella *effectors, so that prediction scheme is somewhat more advantageous compared with our external validation method in the cross-species prediction. Hence, we confirmed that our prediction system can also be applied to the novel genome by comparable accuracy to that of existing tools. Moreover, combinatorial use of our system with a motif based prediction or homology search approach should provide an effective means for screening a *de novo *sequenced genome with no known effectors.

**Table 2:** Predictive powers for the various combinations of feature values in the LT2 validation

	AUC	RANKavg	# of TPs in top 20
ALL	0.993	40.5	9.7
ALL-Nterminal Stability	0.989	57.9	7.6
ALL-CAI	0.991	48.4	8.9
ALL-ProtParam	0.989	56.7	7.9
ALL-dN/dS	0.992	43.9	9.1

Average AUC values are shown for the four sets of validation models. The RANKavg column shows the average rank of known effectors for 10 validation sets. The average number of true positives from 20 known testing examples in the top 20 is shown in the right column.

To examine the impact of the individual feature values, we extracted five sets of feature values and assessed the AUC and RANKavg for each of them. Removal of the POODLE-S index from the feature matrix decreased the average AUC values from 0.993 to 0.989, and the RANKavg value increased from 40.5 to 57.9. The second parameter set showing a notable contribution to discriminative power refinement was the set of physicochemical parameters from ProtParam. In this case, the AUC value was also decreased from 0.993 to 0.989, and the RANKavg value increased from 40.5 to 56.7. Although the CAI parameters showed only moderate differences if they were removed from the discriminant matrix (*e.g.*, a decrease in AUC value from 0.993 to 0.991), we confirmed the statistical significance of these differences. Furthermore, the efficacy of the index was also confirmed by two cross-species prediction models: LT2-to-DC3000, and DC3000-to-LT2 prediction models (See Additional file 3 Supp_Table_CrossPred.xls). The importance of these three parameters: Poodle-S, CAI, and ProtParam, was also confirmed by the decrease in average true positive counts in the top-20 from 9.7 to 7.6, 8.9 and 7.9 on removal of Poodle-S, CAI, and ProtParam, respectively (Table 2), which corresponded to one or two losses of true positives.

On the other hand, the dN/dS parameter showed a negligible difference if removed from the matrix, though the dN/dS values were estimated to be significantly higher for the effector group than for the proteome. This may be because the feature represented by dN/dS correlates highly with features indicating an alien origin for genes, such as low GC content and low CAI. The insufficiency of orthologous sequences due to the rapid turnover of effector genes could make the dN/dS parameter ineffective. Hence, the inclusion of sequence data from whole genome shotgun reads increases the effective orthologous sequences of some effectors and may further refine the accuracy of our system. The charge and pI value parameters showed different tendencies between LT2 and DC3000. Inclusion of these parameters decreased the discriminant power in the cross-species prediction (Additional file 3 Supp_Table_CrossPred.xls), as expected from the opposite tendencies of the effectors between the two organisms (Table 1 and Additional file 2 Supp_Table_StatDC3000.xls).

### N-terminal flexibility prediction method and its impact on effector discrimination

**Figure 1:**
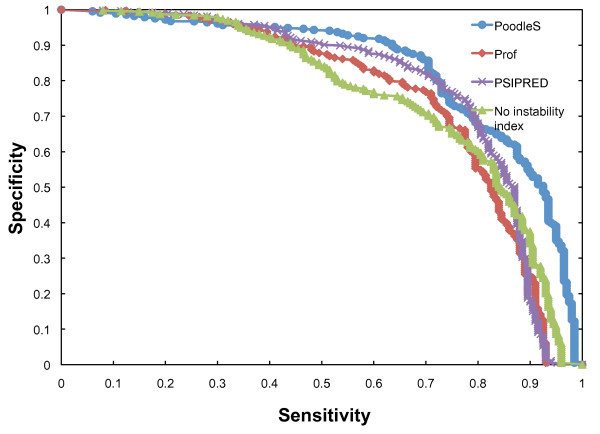
**ROC curves of the performance of different prediction tools for N-terminal instability**. The values for sensitivity and specificity were averaged for ten validation sets. To remove the effect of abundant negative examples, the top 200 ranked genes were selected and the ROC curve was created from these.

Methods for predicting secondary structure from the primary sequence have been developed by many research groups, and prediction power has attained accuracy rates of over 80 percent [30]. This indicates that discrimination among coil, beta-sheet, or alpha-helix structures can be assigned by these methods with a high degree of accuracy. However, the same coil structure regions can have different degrees of flexibility depending on the structural context of the region. A support vector machine (SVM)-based method proposed by Yang and co-workers implemented solvent accessibility criteria for the secondary structure element being investigated and in this way improved the accuracy of effector prediction [18]. In the present study, we assumed that the incorporation of an accurate method for assessing N-terminal flexibility would improve prediction accuracy. We selected POODLE-S for the analysis because this method considers location when the flexibility of amino acid sites is estimated on the basis of primary sequence. The process used to optimize the threshold for a judgment of flexibility at a given site is described in the Additional file (Additional file 4 Supp_Doc_FlexParm.doc). For comparison, we also performed the widely-used secondary prediction programs Prof [31] and PSIPRED [32] for the analysis. Figure 1 shows the recursive operational curve (ROC) of the top-200 ranked of 10 randomly selected validation sets for the LT2 model. We noticed that the incorporation of Prof did not significantly improved prediction accuracy, which agreed with a report by Wang and Arnold [15,16]. In their reports, they concluded that including alphabets of secondary structure prediction results could not refine the prediction accuracy. Using PSIPRED for prediction resulted in a slight refinement of performance, as seen in the upward shift of the ROC. In contrast, the incorporation of POODLE-S for flexibility judgment clearly improved prediction accuracy. Thus, we concluded that incorporating an accurate assessment of N-terminal flexibility certainly improved prediction performance. This result is consistent with a recent report by Buchko and co-workers, which showed that the intrinsically disordered nature of the N-terminal region determines the ability to act as a TTSS substrate [33]. The prediction accuracy of POODLE-S may be less than perfect for estimating the flexibility of the N-terminal region. More accurate assessment of N-terminal flexibility may further improve the overall prediction accuracy. However, the ability to predict flexibility from primary sequence information alone may be limited. Flexibility assessment by methods based on analysis of the dynamics of protein structure in three-dimensional space, such as molecular dynamics simulation, is a promising way to improve accuracy. The integration of molecular dynamics simulation for effector prediction is currently under investigation in our laboratory.

### N-terminal codon usage of effectors is de-optimized

The inclusion of CAI in the SVM-based prediction strategy had a limited effect on the refinement of overall prediction accuracy. One reason is that the feature of low CAI is partially correlated with low GC content, which is a symptom of alien origin. However, we have considered that the low CAI feature may stem from other aspects of effectors besides alien nature. To investigate codon bias in the N-terminal regions of the effectors, we compared the N-terminal CAI with the entire CAI. As a result, the CAI of the known effectors for the 25 N-terminal aa sites showed a significantly lower average value (0.53) in the LT2 model than that (0.57) for the entire protein sequence (Student's T-test, p-value 0.002). To investigate the positional difference of the bias, the ratio of the entire CAI to the N-terminal CAI was estimated for all effectors and for all other genes. There were 32/40 (80.0%) cases of known LT2 effectors in which the ratio exceeded 1. To compare values in non-effector genes with alien origins, we selected genes with similar GC content values to those of known effectors. A total of 651 genes with GC contents between 0.38 and 0.48 were selected. Of these, the ratio exceeded a value of 1 in 395 (60.7%) cases. The number of cases in which the ratio exceeded 1 in the effector group was significantly greater than that in the low GC genes (Fisher's exact test, p-value = 0.0094). We also performed window analysis of non-optimal codon usage in the N-terminal region and found that codon de-optimisation was especially prominent in the region between 1 and 32 in the group of known effectors (See Additional file 5 Supp_Doc_CAI.doc). De-optimisation was more prevalent in the known effectors than in putative alien genes. Interestingly, the distribution of non-optimal codon usage in the N-terminal region showed a similar tendency to that of the putative substrate of the Sec translocon. Kampenusa *et al. *recently reported that the CAI was useful for discriminating among substrates from four different types (I, III, IV, and VI) of secretion systems [34]. The present study revealed that codon bias was especially prominent in the N-terminal region of the secretion substrates. Therefore, codon de-optimisation may stem from a specific translocation mechanism. This characteristic has also been described for the substrate of the Sec-dependent translocon [25,26]. One possibility is that slow translation of the secretion substrate may be needed for efficient co-translational translocation or for protection against the proteolytic degradation of proteins with disordered N-termini.

### Increase in enrichment of known effectors in the top ranking by secondary filtering

**Figure 2:**
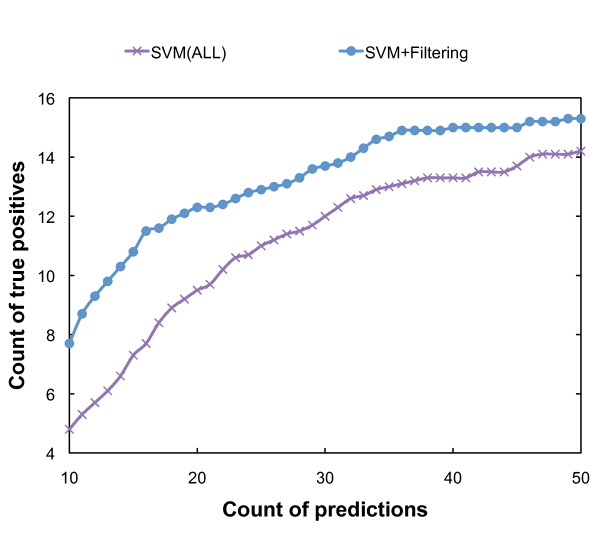
**Refinement of predictive power by additional filtering in the LT2 internal model**. 'SVM(ALL)' represents the distribution of true positive counts as a function of the count of predictions for top-50 ranking in SVM portion of the analysis. The true positive count of 'SVM+Filtering' was re-estimated for prediction ranking after simple-criteria-based filtering. The true positive count for the respective total prediction count was the average of 10 randomly selected validation sets.

Our SVM-based machine learning approach showed robust prediction power upon incorporation of several new features as mentioned above. However, the prediction power was still insufficient, since the average rank of known effectors deviated from the optimal value. For example, among 10 randomly selected validation sets, the average effector rank showed a top value of 27.9 for Set1. This value is still higher than the optimal score of 10.5 for 20 known effectors, assuming no novel effectors had been uncovered. Moreover, apparent false positives such as virulence-related transcriptional regulators were partially contained in the top ranking of the prediction. The top ranking also contained virulence proteins related to glycome metabolism. These proteins showed low GC content and other alien gene-like features. We considered the possibility that it is simply difficult to eliminate these virulence proteins, which resemble true secretion substrates, using the SVM approach. Therefore, we conducted a further filtering process to eliminate these false positive cases. The criteria for the final filtering process consisted of coexpression analysis, composition of negatively charged residues (Asp, Glu) in the first 15 aa, CAI, ORF length, domains commonly seen in bacteria (domain distribution analysis), and a search for similarity to apparatus proteins. Apparatus proteins are thought to be conserved among different strains or even distant organisms because the secretion systems of many organisms have a common origin. Similar architecture among these organisms has been adapted, as exemplified by the exchangeable ability to translocate heterogeneous substrates from different organisms [35]. Therefore, apparatus proteins are expected to be removed easily by homology search. In addition to this simple filtering process, we incorporated a coexpression index with known effectors for further filtering. The expression of effectors is strictly controlled by multiple regulatory systems to ensure that they function at the appropriate times during infection [36,37]. For example, the *Salmonella *Pathogenesity Island (SPI)-2 effectors are regulated by the two-component system SsrA/B, which senses the intracellular environment inside a macrophage [38]. The pattern of expression of effectors is expected to be different from that of housekeeping genes, which are generally constitutively expressed. We confirmed that the expression patterns of known effectors, estimated by the assembly of microarray experimental data in the gene expression omnibus (GEO) database, correlated with each other (See Additional file 6 Supp_Table_CoEXP.xls). The filtering threshold was optimized as described in the Methods section. As shown in Figure 2, the introduction of secondary filtering in the LT2 model further increased the enrichment of true positive cases for the same number of predictions. The number of true positives from 20 known effectors in the top 20 ranking reached 12.3 on average. Hence, our system has good predictive power that is sufficient for candidate selection, which should then be followed by thorough, gene-targeted experimental verification. Incorporation of these filtering indices into SVM matrices also refined the prediction results at a similar level to that seen with step-wise filtering. However, we also adopted heuristic filtering after the SVM analysis, because independent coexpression analysis provided very important information regarding to the regulatory network of a given gene (*e.g*. co-regulated with SPI-1 or SPI-2 genes). Refinement resulting from the additional filtering was also seen for the dataset of DC3000 (See Additional file 7 Supp_Doc_SecFilDC3000.doc).

Secondary filtering by coexpression analysis can be applied only to organisms with at least one expression dataset deposited in a public database. Hence, this filter cannot be applied in the vast majority of *de novo *assembled genomes. Although we assembled 11 and 4 expression datasets from LT2 and DC3000, respectively, enrichment of true positives in the top-ranking of the first part of the SVM analysis can be improved with as little as one expression dataset with 10 or more sample slides (See Additional file 8 Supp_Doc_ExpRequirement.doc). Since it is expected that the deposition of expression data will increase at a rapid rate owing to the tremendous progress in next-generation sequencing technologies (*e.g*. RNASeq whole cell transcriptome analysis), heuristic filtering by coexpression analysis will be more feasible in the future screening of virulence effectors.

### Putative model effectors predicted by this system and further assessment by recently found effectors

Since the identification of *Salmonella *effectors may be still under way, the putative list predicted by our system is expected to contain many novel effectors. To predict putative effectors, the prediction was performed again using all of the known effectors as the training set and optimized by negative set selection. As mentioned above, the AUC value among the 10 randomly selected validation sets showed the highest score for Set1, which indicates that the selection of negative examples in Set1 is optimal for the discrimination analysis. Using this set of negative samples, SVM prediction was performed, followed by additional filtering. The putative novel effectors predicted by this analysis are listed in Table 3. If there are no additional effectors in the genome and the prediction accuracy is optimal, the known effectors should all be listed in the top 40. In this analysis, 36 out of 40 known effectors were predicted in the top 40, corresponding to 90% sensitivity and 90% specificity. Two putative effectors were predicted in top-10. One is STM1055 (*gtgE*), which was recently reported to be an effector by Niemann and co-workers, who examined translocation into macrophages by the CyaA assay [10]. The other protein, encoded by *pipA *and judged by SIEVE analysis to be a putative effector, was predicted at the 3^rd ^rank. McDermott and co-workers examined the translocation of this protein and reported that it showed no indication of translocation in their CyaA assay [39]. Although this result indicates that *pipA *is not an effector, it is possible that the protein is actually translocated into host cells under specific conditions (*e.g*. functional only inside specific cell type or activated by some stimuli). Besides *gtgE*, Niemann *et al. *recently revealed five additional effectors through a liquid chromatography-mass spectrometry (LC-MSMS)-based proteome experiment. The two effectors STM1026 and PSLT039 were treated as known effectors in our validation and successfully ranked at the 69^th ^and 24^th ^respectively. In our prediction ranking, two other TTSS dependent effectors identified by Niemann and co-workers, STM2139 and STM2585, ranked 48^th ^and 56^th^, respectively, which correspond to 12^th ^and 20^th ^after removal of known effectors from the ranking. Another TTSS-dependent effector, STM3762 (CigR), was discarded in our prediction owing to a lack of effector-like features; it was predicted to have an unstable N-terminus, but the CAI, phylogenetics, and other features showed housekeeping-like characteristics. For example, the gene has been predicted to have orthologs in *Klebsiella*, which are thought not to have TTSS apparatus systems. In spite of this single false negative case, the fact that three recently-identified effectors, along with the two cases in the gold-standard set, fell within an acceptable range in the gene-targeted experimental assessment supported the feasibility and accuracy of our system for effector prediction. Treatment of exceptional cases, for example STM3762 from the first discriminant analysis, is a point requiring future improvement. Interestingly, the other top ranking non-effectors are mostly hypothetical genes and are annotated as virulence-related (references for virulence annotation are listed in the Additional file 9), which supports the probability that they may in fact be virulence effectors. Experimental verification of these proteins is under way in our laboratory.

**Table 3:** Putative novel effectors of serovar Typhimurium predicted by the system

Rank	STM-ID	Name	Description	PSOTb 3.0	Ref. ^#^	Poodle-S
3	STM1087	*pipA*	pathogenicity island encoded protein: SPI3	Cytoplasmic	[10]	13

4	STM1055	*gtgE*	Gifsy-2 prophage	Unknown	[6]	17

32	STM3155		putative cytoplasmic protein	Unknown	[6]	18

40	STM1239		putative cytoplasmic protein	Cytoplasmic	-	11

41	STM2534		putative cytoplasmic protein	Cytoplasmic	-	12

42	STM2007		putative TPR repeat protein	Extracellular	[2]	7

43	STM1670		putative serine/threonine protein kinase	Unknown	[1,2,6]	16

44	STM3278		putative cytoplasmic protein	Unknown	[3]	9

45	STM2761		putative inner membrane protein	Cytoplasmic	[3]	10

46	STM4504		putative cytoplasmic protein	Cytoplasmic	[2]	3

47	STM0335		putative outer membrane protein	Unknown	-	4

48	STM2139	*steD*	putative inner membrane protein	Unknown	[2,5]	24

49	STM2779		putative inner membrane protein	Unknown	-	21

50	STM1554		putative coiled-coil protein	Unknown	[1]	13

51	STM4302		putative cytoplasmic protein	Unknown	[3]	15

52	STM1939		putative glucose-6-phosphate dehydrogenase	Unknown	[1,2]	13

53	STM3052		putative outer membrane protein	Unknown	[1]	8

54	STM2879	*sicP*	chaparone, related to virulence	Cytoplasmic	-	9

55	STM0497		putative periplasmic protein	Unknown	-	10

56	STM2585	*steE*	Gifsy-1 prophage: similar to transpose	Unknown	[1]	12

57	STM2008		putative periplasmic protein	Unknown	[1,2]	8

58	STM2893	*invI*	Surface presentation of antigens; secretory proteins	Cytoplasmic	[1-3,6]	10

59	STM1669		Homology to invasin C of *Yersinia*; intimin	OuterMembrane	[1,6]	9

60	STM1940		putative cell wall-associated hydrolase	Unknown	-	14

#---References for virulence annotation from the high throughput experiments. The list of references is described in Additional file 9 'Supp_Table_VirlenceAnnot.xls'.

Because the exact number of unidentified effectors in the genome is unknown, it is possible that the highly ranked not-known-effector genes are actually true effectors. Hence, the enrichment of known effectors in the top ranking only does not indicate the predictive performance. This is the main difficulty in assessing the performance of effector prediction. However, the high rankings for recently identified effectors, taken together with the enrichment of known effectors in our validation set, suggest that the results of our prediction approach a complete catalogue of effectors; at least, we could make an almost-complete candidate list for identifying effectors that have common characteristics with known TTSS substrates.

In the present study, we confirmed that our system showed significant improvement over existing methods and revealed several novel discriminant features. However, not all of the revealed features, including previously reported ones, were specific enough to precisely determine the substrate, *i.e*. were clear recognition signals. Construction of prediction tools should support the deciphering of the recognition mechanisms of the secreted proteins through the implication of specific recognition signals or precise recognition principles. It is speculated that such signals, related to translocation mechanisms, are encoded at the three-dimensional level, especially considering the failure to detect a common motif in the primary sequence of TTSS substrates, in spite of recent advances in prediction tools. The use of structural informatics to further refine the prediction system is considered to be a promising approach for the future development of prediction tools.

## Conclusions

We developed a meta-analytic approach to predicting virulence effectors accurately by integrating discrimination features derived from the genome sequence information and DNA microarray experimental data. Our analysis consisted of two parts. The first, based on SVM learning, is an approach developed through modification of existing tools. In this SVM-based analysis, new parameters were introduced as follows: (i) N-terminal flexibility estimation by the POODLE-S program, (ii) structure-related parameters estimated by ProtParam, such as grand average of hydropathicity(GRAVY score), and (iii) codon adaptation index. The introduction of these new parameters refined the discriminatory power of the tool. The use of N-terminal flexibility as a determinant of TTSS substrate status has been controversial. In this study, we confirmed that the incorporation of accurate assessment of N-terminal flexibility genuinely refined the prediction power, which supports the hypothesis that N-terminal flexibility is an important feature of the TTSS substrate. The second part of our analytical framework, additional filtering through coexpression analysis using the DNA microarray data in the GEO database and functional domain distribution analysis, further refined the predictive power of our system. In our benchmark test, the number of true positives from 20 known effectors in the top 20 ranking reached 12.3 on average. Hence, our system has good predictive power that is sufficient for candidate selection, which should then be followed by thorough, gene-targeted experimental verification. Furthermore, the putative effectors predicted by our system in the LT2 contained many hypothetical genes and genes with virulence annotation, indicating additional novel effectors in the *Salmonella *genome. In addition to the successful construction of this system, we also revealed intriguing features of effectors, namely that N-terminal codon usage is significantly de-optimized and that the N-terminal region is predicted to take on a highly flexible structure in these proteins.

## Methods

### Construction of the gold-standard validation set

Known effectors of *Salmonella enterica *serovar Typhimurium LT2 and *P. syringae *DC3000 were assembled on the basis of the literature (See Additional file 1 Supp_Table_knownEffector.xls). In total, 40 and 28 known effectors were annotated for LT2 and DC3000, respectively. The annotation data for all 4550 protein-coding genes for LT2 and 5619 protein-coding genes for DC3000 were downloaded from the KEGG GENES database [40]. All genes except for known effectors were treated as negative examples in the validation process. To assess the accuracy of prediction, all positive and negative examples were separated into two sets, the training set and the test set. The ratio of positives to negatives in the training set was set at 1:20, according to the study by Samudrala [17]. The training set was used for the learning of discrimination features and the trained discrimination device was then applied to the rest of the known effectors in the test set. All of the negative examples other than known effectors were included in the test set. The prediction accuracy is highly dependent on the selection of the negative training set. Therefore, 10 sets of training and testing examples were randomly selected (Set1 ~ Set10) and the AUC values were averaged.

### Accurate annotation of the translation initiation site

Because the signal in the N-terminal region is also one of the important features in our prediction system, the effect of translation initiation site accuracy for effector prediction was first assessed. The translation initiation sites (TISs) were re-annotated by integrating three different TIS annotations: those from the KEGG database, ProTISA, and geneFinder. The TISs of two known effectors, STM1026 and STM2088, were re-annotated. Experimentally validated TISs for these two effectors have not yet been revealed. Regarding STM1026, our previous experiment assessing the translocation of STM1026 suggests that the translocation of this effector requires a re-annotated and elongated, *i.e.*, upstream, ORF (our recent result, Takaya *et al., submitted*). As for the ORF annotation of DC3000, such incorrect annotation was not observed for known effectors. Therefore, annotation from the KEGG GENES database was used for the SVM analysis for DC3000.

### Coexpression analysis using microarray data of GEO datasets

Transcriptome profiling datasets were assembled and all-versus-all comparisons of gene expression similarity were conducted. Eleven and four datasets were selected for the analysis of LT2 and DC3000, respectively (See Additional file 10 Supp_Table_GEODataSet.xls). Firstly, expression data were downloaded from GEO and were normalized to one dataset by Z-score. The similarity of expression profiles between two genes was calculated by Pearson correlation. The expression similarity scores (correlation coefficients) against the selected known effectors from the training set were averaged and defined as a score of coexpression with the known effectors. In serovar Typhimurium, the expression of effector proteins is controlled by two different systems, SPI-1 and SPI-2. Therefore, the effector proteins were divided into two groups according to the regulatory system, and coexpression analysis was conducted separately. The coexpression pattern was clearly observed among known effectors of LT2 (Additional file 6 Supp_Table_CoEXP.xls). Almost all of the known effectors ranked in the top 300 in the SPI-1 or SPI-2 assessment, suggesting that coexpression analysis could successfully discern the tendency of effector genes to be coexpressed.

### Estimation of each parameter used in the SVM analysis

Molecular weight, charge, predicted pI, instability index, aliphatic index, and GRAVY score were calculated using the ProtParam web server [41]. The instability index of the 25 N-terminal aa was estimated using the POODLE-S web server. The "missing residues" parameter was used for the analysis. For codon adaptation index (CAI), the reference codon tables for serovar Typhimurium (Esty.cut) and *P. syringae *DC3000 (Epsesm.cut) prepared in EMBOSS suite version 2.5.0 [42] were used. The dN/dS ratio was estimated against the multiple alignment of orthologous genes annotated from all available *Salmonella *genomes in the KEGG database. The codeml module of the PAML package, version 4.2, was used for the analysis [43]. Amino acid composition, position specific matrix, and phylogeny matrices were estimated as described in SIEVE [17]. As for the phylogeny matrices, several points were modified. A total of 1172 organisms from the KEGG ORGANISMS database were used for the analysis. The identity values for assigned orthologous genes were treated as parameters for 1172 dimensions. If orthologous gene was not assigned for the given organism, the value was set to zero. The reciprocal orthology annotation in KEGG Sequence Similarity Database (SSDB), which is a database of orthologous gene annotation, was used. The organisms used for the analysis are listed in the Additional file 11 (Supp_Table_Organisms.xls). The parameters used in the SVM analysis are summarized in the Additional file 12 (Supp_Table_FeatParms.xls).

### Validation set construction, kernels and parameters used in the SVM analysis

In this study, validation was performed on a genome-wide scale. Therefore, the test set consisted of all of the genes from the respective genomes. The training set consisted of a randomly selected positive set with half of the known effectors. The remaining half was used for validating the prediction power. The negative examples for the training set were selected randomly from the proteome. The area under the curve (AUC), the average true positive count in top 20 ranking and the mean rank of the positive examples averaged for 10 validation sets (we call the score RANKavg) were estimated and were used to assess accuracy. The radial kernel function with width factor of 1.0 was used according to the benchmark test for LT2 model (See Additional file 13 Supp_Doc_KernelOptimisation.doc). The seed for the random value was set to 76543. Other parameters were set to default values. SVM analysis and ROC estimation were performed using the Gist package 2.3 [44].

### Determination of thresholds for secondary filtering

In the second round of the filtering process, five parameters with continuous values were used: ORF length, CAI, ratio of negative charge residues in the first 15 aa, dN/dS value, and coexpression ranking. For optimal thresholds, all combinations of two points for all five parameters were considered. One is the threshold covering all of the positive examples in the respective training set. The second is the value allowing one dropout. The optimal set of thresholds showing the best performance was determined from all combinations of these thresholds. In the second round of filtering, we wanted to filter out non-effector genes in the top ranking, while maintaining the lowest possible dropout rate of known effectors. To accomplish this, the following performance index, which weights recall over precision, was used:

$$\begin{array}{c} \text{Performance}=\left(\text{2}\times \text{Recall}+\text{Precision}\right)∕\text{2}\\ \text{Precision}=\text{TP}∕\text{TP}+\text{FP}\\ \text{Recall}=\text{TP}∕\text{TP}+\text{FN} \end{array}$$

(TP: true positive, TN: true negative, FP: false positive, FN: false negative.) The threshold values determined by the above process were applied to the respective testing examples. Genes satisfying all criteria (AND criteria) were judged as effectors, and others were discarded from the list. An example of the optimal thresholds determined on the basis of the first validation set (Set1) for LT2 is listed in the Additional file 14 (Supp_Table_OptimThresh.xls).

### Annotation of functional domains commonly observed in bacteria

The functional domain annotation and links to the InterPro database were obtained from the KEGG GENES database. The domain distribution information was extracted from the InterPro database. If the given domain annotation appeared over 5000 times in the bacterial species, the domain was judged to be a commonly conserved domain among bacteria. The threshold of 5000 was selected to cover all of the known effectors.

### BLAST homology search for the removal of secretion apparatus genes

First, a set of all secretion apparatus proteins from the KEGG GENES database was collected by keyword search. The keywords used were "apparatus AND secretion AND type" to select genes of all types of secretion apparatuses. In total, 599 genes were selected by the search. To avoid a simple self-hit, all *Salmonella *and *Pseudomonas *genes were removed from the list. In the BLASTP search, if the protein had any hit with an e-value less than 10^-5^, the protein was judged to be an apparatus protein.

### Avairability

The SVM portion of the prediction system is implemented by the wrapper program, which integrates various bioinformatics tools running on the Unix platform and various web application programming interfaces (APIs). The wrapper program is written in Perl and can be run on a Unix-based machine. The source code is available from our web site http://www.p.chiba-u.ac.jp/lab/bisei/software/index.html. The user-friendly web-server version of the prediction system is now under construction and will be available at the above site in the near future.

## Authors' contributions

YS, AT and TY conceived, designed and managed the study. YS designed the prediction system, collected the data, performed the analysis, and drafted the manuscript. All authors read and approved the final manuscript.

## Supplementary Material

Additional file 1 **Supp_Table_knownEffector.xls**. The list of known effectors for LT2 and DC3000. Known effectors used for assessing prediction accuracy are listed. Click here for file

Additional file 2 **Supp_Table_StatDC3000.xls**. Statistics for discrimination features in the model of *P. syringae *DC3000. Means and standard deviations were estimated for the group of known effectors and the rest of the proteome. Click here for file

Additional file 3 **Supp_Table_CrossPred.xls**. Predictive powers for the various combinations of feature values in the DC3000 validation and the LT2-DC3000 cross species validations. To examine the feasibility of our system for another Gram-negative bacteria, the same validation as LT2 was applied to all genes of DC3000. Additionally, cross-species prediction was assessed, *e.g*. the known effectors of LT2 were used for the training and all the known effectors and non-effectors of DC3000 were used for the test and for the accuracy assessment. Click here for file

Additional file 4 **Supp_Doc_FlexParm.doc**. Conversion of POODLE-S output to SVM index. To count the number of flexible sites in the N-terminal region, we used the probability of missing site estimated for each amino acid site by Poodle-S. The threshold value for the judgement of flexibility was optimised by the benchmark test, which estimated the discriminant power of the flexibility score. Click here for file

Additional file 5 **Supp_Doc_CAI.doc**. Non-optimal codon usage in the N-terminal region of effectors. Window analysis calculating optimal codon ratio in the N-terminal region was performed to reveal the characteristic codon usage of the effectors. Click here for file

Additional file 6 **Supp_Table_CoEXP.xls**. Coexpression result for known effectors of LT2. Efficacy of the coexpression analysis to reveal the regulatory network of a given gene was assessed by the all known effectors of LT2. Click here for file

Additional file 7 **Supp_Doc_SecFilDC3000.doc**. Effect of secondary filtering in the DC3000 model. Refinement of predictive power by additional filtering was assessed in the DC3000 model. Click here for file

Additional file 8 **Supp_Doc_ExpRequirement.doc**. Requirement of expression data for effective secondary filtering. Refinement of predictive power by coexpression analysis was assessed by the respective dataset in the 11 and 4 datasets from LT2 and DC3000, respectively. Click here for file

Additional file 9 **Supp_Table_VirulenceAnnot.xls**. References for virulence annotation from high-throughput experiments Click here for file

Additional file 10 **Supp_Table_GEODataSet.xls**. The list of GEO datasets. GEO datasets used for the coexpression analysis of LT2 and DC3000 are listed. Click here for file

Additional file 11 **Supp_Table_Organisms.xls**. List of organisms from KEGG ORGANISMS database used for phylogenetic profiling in SVM analysis Click here for file

Additional file 12 **Supp_Table_FeatParms.xls**. Feature parameters used in the SVM analysis. Click here for file

Additional file 13 **Supp_Doc_KernelOptimisation.doc**. Optimisation of kernel type used for the first round of discriminant analysis. The kernel type and the parameter used for the SVM analysis affects prediction accuracy. SVM analysis with various kernel types were performed and the best kernel was determined based on the AUC of LT2 validation model. Click here for file

Additional file 14 **Supp_Table_OptimThresh.xls**. Example optimal parameters used for secondary filtering of the first validation set (Set1). Optimised thresholds used for the secondary simple-criteria-based filtering in the first validation set (Set1) are listed. Click here for file
