# Supplementary material for: Meta-analytic approach to the accurate prediction of secreted virulence effectors in gram-negative bacteria
Source: BMC Bioinformatics. 2011 Nov 14;12:442. doi: 10.1186/1471-2105-12-442 (PMC3240867; doi:10.1186/1471-2105-12-442)
Supplement: Additional file 4 — Supp_Doc_FlexParm.doc. Conversion of POODLE-S output to SVM index. To count the number of flexible sites in the N-terminal region, we used the probability of missing site estimated for each amino acid site by Poodle-S. The threshold value for the judgement of flexibility was optimised by the benchmark test, which estimated the discriminant power of the flexibility score. [file 1471-2105-12-442-S4.DOC]

**Additional file Supp_Doc_FlexParm.doc**

**Conversion criteria of POODLE-S output to SVM index**

The index of flexibility of the N-terminal region was estimated from the output of POODLE-S. The POODLE-S program outputs the probability of missing residues for each amino acid site. Since the missing residues in the crystallography are caused mainly by high flexibility, this prediction value can be translated to the index of flexibility. To convert these values into a single-dimension parameter for SVM analysis, we set the threshold for probability of missing residue and summed up the number of flexible sites (judged as missing site by above threshold) for the N-terminal at 25 sites. To optimize the threshold, the F-measure was estimated for the respective threshold when known effectors of serovar Typhimurium and 4,510 non-effector genes were treated as positive and negative examples, respectively. It was revealed that a threshold of 0.5 provided the maximum F-measure separating known effectors and non-effectors in a single usage of POODLE-S.


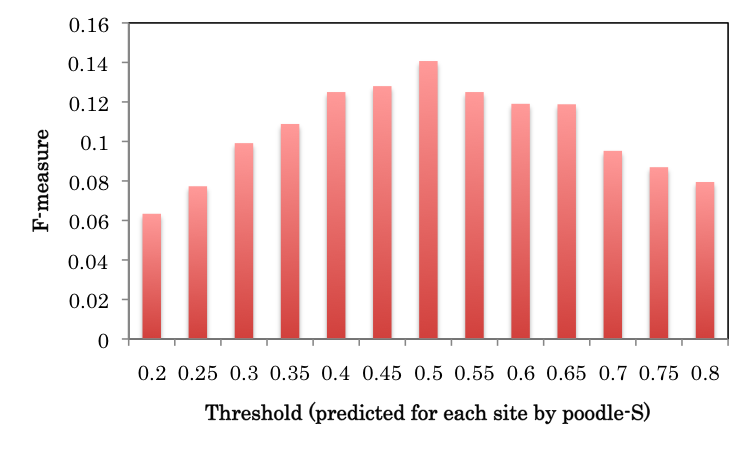


**Fig. S1 Optimisation of the threshold for assignment as a flexible site, from the output of POODLE-S.**
