# Supplementary material for: Meta-analytic approach to the accurate prediction of secreted virulence effectors in gram-negative bacteria
Source: BMC Bioinformatics. 2011 Nov 14;12:442. doi: 10.1186/1471-2105-12-442 (PMC3240867; doi:10.1186/1471-2105-12-442)
Supplement: Additional file 5 — Supp_Doc_CAI.doc. Non-optimal codon usage in the N-terminal region of effectors. Window analysis calculating optimal codon ratio in the N-terminal region was performed to reveal the characteristic codon usage of the effectors. [file 1471-2105-12-442-S5.DOC]

**Additional File Supp_Doc_CAI.doc**

**Non-optimal codon usage in the N-terminal region of effectors**

The fraction of optimal codon usage was estimated for effectors and compared with alien cytoplasmic genes (*i.e.,* genes acquired by horizontal gene transfer), non-alien cytoplasmic genes, and putative Sec substrates. The putative alien cytoplasmic genes were defined as genes with cytoplasmic location prediction by PSORTb3.0 and with GC content between 0.38 and 0.48, assuming similar distribution of GC content with known effectors. As for the set of putative Sec substrates, genes were selected for which subcellular location was predicted or annotated as the outer membrane by PSORTb version 3.0. The codons showing codon frequencies of less than 0.3 were considered non-optimal because all amino acids have at least one optimal codon with a frequency over 0.3 in the dataset of LT2. The codon frequency data used were as annotated in Esty.cut of EMBOSS 2.6. As shown in the Fig. S2, the fraction of non-optimal codons of the effector group was higher in the N-terminal region. The set of outer membrane proteins also showed a similar tendency but had more optimised codons in the downstream region. Cytoplasmic proteins from proteome and putative alien genes showed a gradual increase in non-optimal codon usage in the N-terminal region, which may simply reflect annotation errors regarding TISs. However, these two sets did not represent a prominent increase in non-optimal codon usage in the N-terminal region as seen in the putative Sec substrate or known effectors. Hence, this supports our proposal that the N-terminal codon adaptation index was significantly de-optimized in the effector group, as described in the main text.


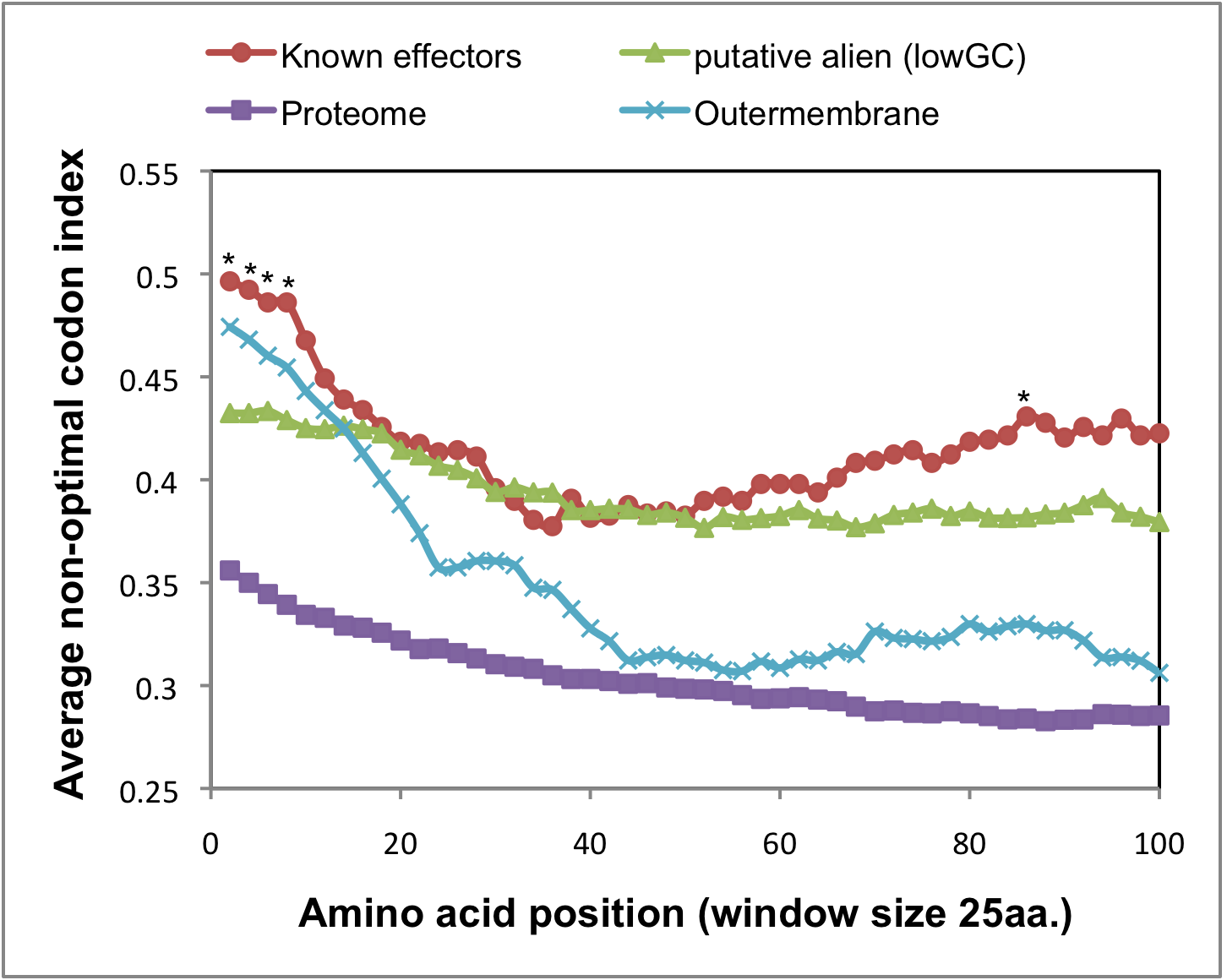


**Figure S2 Codon usage in the N-terminal region of effectors is de-optimized.**

The sites showing statistically significant difference (Student T-test, p-value ≤ 0.01) between putative alien cytoplasmic genes and known effectors, are marked by asterisks.
