# Supplementary material for: Meta-analytic approach to the accurate prediction of secreted virulence effectors in gram-negative bacteria
Source: BMC Bioinformatics. 2011 Nov 14;12:442. doi: 10.1186/1471-2105-12-442 (PMC3240867; doi:10.1186/1471-2105-12-442)
Supplement: Additional file 7 — Supp_Doc_SecFilDC3000.doc. Effect of secondary filtering in the DC3000 model. Refinement of predictive power by additional filtering was assessed in the DC3000 model. [file 1471-2105-12-442-S7.DOC]

**Additional File Supp_Doc_SecFilDC3000.doc**

**Effect of secondary filtering in the DC3000 model**


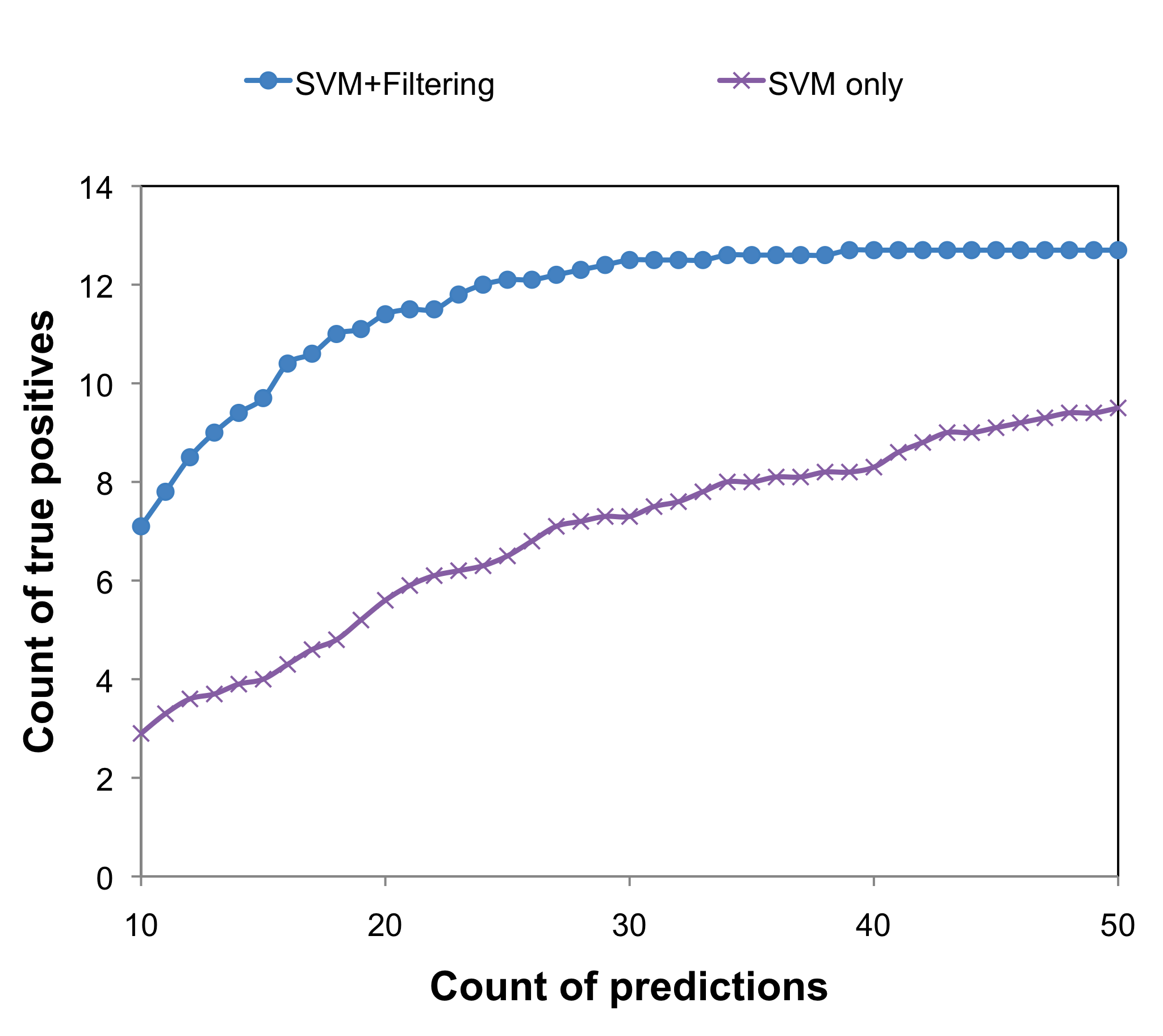


**Figure S3 Refinement of predictive power by additional filtering in the DC3000 model.** **The count of true positives as a function of the count of prediction is shown for top-50 ranking of DC3000 internal prediction model.** ‘SVM+Filtering’ represents the distribution for re-ranked prediction after secondary filtering by coexpression analysis as described in LT2 prediction. ‘SVM only’ represents the distribution of SVM portion of analysis.
