# Supplementary material for: Meta-analytic approach to the accurate prediction of secreted virulence effectors in gram-negative bacteria
Source: BMC Bioinformatics. 2011 Nov 14;12:442. doi: 10.1186/1471-2105-12-442 (PMC3240867; doi:10.1186/1471-2105-12-442)
Supplement: Additional file 8 — Supp_Doc_ExpRequirement.doc. Requirement of expression data for effective secondary filtering. Refinement of predictive power by coexpression analysis was assessed by the respective dataset in the 11 and 4 datasets from LT2 and DC3000, respectively. [file 1471-2105-12-442-S8.DOC]

**Supp_Doc_ExpRequirement.doc**

**Required DNA microarray data for effective coexpression analysis**

We have assembled 11 and 4 datasets of DNA microarray experiments from LT2 and DC3000, respectively, to conduct heuristic filtering by coexpression analysis. The filtering process successfully filtered out non-effector proteins in the top-ranking of the first SVM analysis. Although we could perform the successful coexpression analysis by using sufficient amount of expression data for LT2 and DC3000, most of the organisms other than the deeply investigated model organisms have only one or no expression datasets deposited in GEO. Thus, it is unclear that the coexpression analysis is effective for such organisms with a few (one or two) expression datasets. To reveal the required amount of expression data for effective filtering by coexpression analysis and the suitability of each expression experiment for this analysis, we investigated the efficacy of filtering by the respective dataset in 11 datasets of LT2 and 4 datasets of DC3000.

In our SVM analysis, most of the known effectors in a gold-standard-set were predicted at worst within top-200. Successful removal of non-secreted proteins from top-200 by coexpression filtering will help effective screening by use of the prediction result. Therefore, we estimated the odds ratio to assess the filtering power of the respective dataset, as follows:

Odds ratio = ( A / B) / (C / D)

A = The number of true positives in top-200

B = 200 - A

C = The number of false negatives in discarded genes by the coexpression filter

D = The number of discarded genes by the coexpression filter

Each value was averaged from 10 validation sets as described in the main text. In the LT2 validation, four known effectors without co-regulation information with SPI-1 or SPI-2 genes (refer to additional file 6) were removed in this analysis. Fisher’s exact test was used to test whether or not the enrichment of the effectors in the genes discarded by filtering was significantly reduced compared to that in top-200 prediction.

As a result, three datasets from 11 LT2 datasets showed significant improvement for the true positive rates (Table SI-1). In the case of DC3000, two of four datasets showed significant improvement for the true positive rate (Table SI-2). The GEO datasets, which showed an effect of improvement, consist of from 9 to 32 sample slides. Hence, only one dataset of DNA microarray experiment can assist the screening of virulence effectors. As shown in the case of 11^th^ dataset of LT2 and 4^th^ dataset of DC3000, microarray experiments to investigate regulatory mechanisms related to TTSS showed strong discriminant power to filter out irrelevant genes. Intriguingly, expression profiling without apparent connection with virulence or TTSS regulatory network also showed efficacy to pick up virulence effectors as shown in the case of microarray experiment to reveal iron regulation in *P. syringae* DC3000 (dataset 2). Furthermore, we also revealed that assembling of different expression experimental data showed the best performance to filter out false positive genes in the top-ranking.

Table SI-1 Discriminant power to filter out non-effector proteins for respective GEO dataset of LT2

| GEO datasets | Title | Odds ratio | P-value | Avg. FP cases | Avg. # of discarded |
| --- | --- | --- | --- | --- | --- |
| Assembly | Dataset 1 to 11 | 9.558 | 8.782E-20 | 0.9 | 88.6 |
| 1 | Transcriptional profiles of *Salmonella* typhimurium wt, hfq and smpB mutants in four different growth conditions | 3.398 | 1.443E-03 | 0.5 | 17.5 |
| 2 | IAH *Salmonella* infected chicken caecal contents | 1.467 | 7.772E-02 | 1.8 | 27.2 |
| 3 | Effect of ppGpp on *Salmonella* Typhimurium gene expression | 1.352 | 1.905E-01 | 1.3 | 18.1 |
| 4 | Expression analysis of hns mutant Salmonella | 1.443 | 1.213E-01 | 1.4 | 20.8 |
| 5 | 14028 time course on non-swarming plates | 0.796 | 7.795E-01 | 0.5 | 4.1 |
| 6 | Effect of LacI (lac repressor) on transcription in Salmonella | 1.651 | 9.312E-02 | 0.9 | 15.3 |
| 7 | PreA overexpression in *Salmonella* typhimurium | 0.735 | 8.389E-01 | 0.7 | 5.3 |
| 8 | TL2_14028s_M9+arabinose_LibAra_vs_Lib0 | 1.315 | 2.453E-01 | 1.1 | 14.9 |
| 9 | Effect of hfq-deletion on *Salmonella* transcriptome | 5.670 | 7.765E-07 | 0.5 | 29.2 |
| 10 | Gene Expression profiling of *Salmonella* typhimurium Wild Type and HRG Mutant under H2O2 Stress | 2.265 | 1.081E-01 | 0.3 | 7 |
| 11 | *Salmonella* typhimurium igaA(T191P) and related | 8.269 | 2.419E-12 | 0.6 | 51.1 |

The average number of true positives in top-200 was 17.7.

Table SI-2 Discriminant power to filter out non-effector proteins for respective GEO dataset of DC3000

| GEO datasets | Title | Odds ratio | P-value | Avg. FP cases | Avg. # of discarded |
| --- | --- | --- | --- | --- | --- |
| Assembly | Dataset 1 to 4 | 9.49 | 9.67E-24 | 1.3 | 176 |
| 1 | The expression profiles of PSPTO_2222 and PSPTO_2222-2223 mutants in MM or KB | 6.20 | 1.35E-15 | 1.4 | 123.9 |
| 2 | Iron regulation in *P. syringae* DC3000 | 5.14 | 2.40E-17 | 2.4 | 175.9 |
| 3 | lonB mutant [*Pseudomonas syringae* pv. tomato str. DC3000] | 1.44 | 0.16 | 1.1 | 22.6 |
| 4 | Expression profiles of hrpRS- and hrpL- mutants | 6.64 | 7.98E-20 | 1.8 | 170.6 |

The average number of true positives in top-200 was 13.1.
