# Supplementary material for: Meta-analytic approach to the accurate prediction of secreted virulence effectors in gram-negative bacteria
Source: BMC Bioinformatics. 2011 Nov 14;12:442. doi: 10.1186/1471-2105-12-442 (PMC3240867; doi:10.1186/1471-2105-12-442)
Supplement: Additional file 13 — Supp_Doc_KernelOptimisation.doc. Optimisation of kernel type used for the first round of discriminant analysis. The kernel type and the parameter used for the SVM analysis affects prediction accuracy. SVM analysis with various kernel types were performed and the best kernel was determined based on the AUC of LT2 validation model. [file 1471-2105-12-442-S13.DOC]

**Additional file Supp_Doc_KernelOptimisation.doc**

**Optimisation of kernel type used for the first round of discriminant analysis**

It has been known that difference of the kernel affects discriminant power of the support vector machine analysis. In the study, accuracy assessment by the gold-standard of LT2 was performed using various kernels. The predictive power for the given kernel was assessed by the average AUC and the average rank of testing known effectors estimated for 10 validation sets as described in the Method section of main text. The average AUC values were estimated to be in the range from 0.991 to 0.993, among different kernels (Table SI-3). For the radial kernel and the diagonal kernel, the AUC values were all estimated to be the best value of 0.993 regardless of the parameters used. The radial kernel together with the widthfactor of 1.0 showed the best average rank of 40.45 among the tested parameters.

**Table. SI-3** Predictive powers for various kernels and parameters for SVM analysis

| Kernel Type | Average AUC | Average rank |
| --- | --- | --- |
| Liner, constant=2 | 0.992 | 46.52 |
| Liner, constant=3 | 0.992 | 45.75 |
| Radial, widthfactor=2 | **0.993** | 42.94 |
| Radial, widthfactor=1.5 | **0.993** | 41.48 |
| Radial, widthfactor=1 | **0.993** | **40.45** |
| Radial, widthfactor=0.5 | **0.993** | 41.78 |
| Power=5 | 0.991 | 53.10 |
| Power=4 | 0.992 | 45.61 |
| Power=3 | 0.992 | 44.23 |
| Power=2 | **0.993** | 44.72 |
| Diagonal, add=3 | **0.993** | 42.98 |
| Diagonal, add=2 | **0.993** | 43.04 |
| Diagonal, add=1 | **0.993** | 43.15 |
